# Do Silicon and Salicylic Acid Attenuate Water Deficit Damage in *Talisia esculenta* Radlk Seedlings?

**DOI:** 10.3390/plants12183183

**Published:** 2023-09-06

**Authors:** Vanda Maria de Aquino Figueiredo, Silvana de Paula Quintão Scalon, Cleberton Correia Santos, Jéssica Aline Linné, Juliana Milene Silverio, Wállas Matos Cerqueira, João Lucas da Costa Santos de Almeida

**Affiliations:** 1 Faculty of Agricultural Science, Federal University of Grande Dourados, Highway Dourados, Itahum/km 1, Dourados 79804970, MS, Brazil; vandam.aquino@hotmail.com (V.M.d.A.F.); cleber_frs@yahoo.com.br (C.C.S.); jessica.aline.linne@gmail.com (J.A.L.); juliana.milene@hotmail.com (J.M.S.);; 2 Federal Institute of Science and Technology of Mato Grosso do Sul, Ponta Porã 79909000, MS, Brazil; joao.almeida@ifms.edu.br

**Keywords:** abiotic stress, pitomba, phenotypic plasticity, photosynthetic metabolism, proline

## Abstract

Water deficit is one of the factors that most influence plant growth and yield. Thus, this study aimed to evaluate the effect of silicon and salicylic acid application and water deficit on the growth and photosynthetic and osmoregulatory metabolism of *Talisia esculenta* Radlk. seedlings and their recovery potential after the resumption of irrigation. Six treatments were performed: irrigation, irrigation suspension, irrigation suspension + silicon at 0.5 g L^−1^, irrigation suspension + silicon at 1.0 g L^−1^, irrigation suspension + salicylic acid at 50 mg L^−1^, and irrigation suspension + salicylic acid at 100 mg L^−1^. The evaluations were carried out at the beginning of the experiment, at 10 and 15 days after irrigation suspension, when the seedlings showed a photosynthetic rate close to zero, and at recovery. The plants were subjected to water restriction for up to 15 days, then re-irrigated until the recovery point, which was monitored based on the photosynthetic rate. Silicon application attenuated the harmful effects of water deficit on gas exchange and initial fluorescence, promoted proline accumulation in the leaf and root, and provided higher seedling quality. Salicylic acid application contributed to the maintenance of the relative water content of leaves during the water deficit period. Silicon and salicylic acid applications can attenuate the harmful effects of water stress, with silicon being the most effective agent in maintaining its growth and metabolism.

## 1. Introduction

*Talisia esculenta* Radlk., (*Sapindaceae*), also known as olho-de-boi, pitomba-do-mato, and pitomba-de-macaco, is a fruit tree native to the Amazon region, found naturally throughout Brazil, is well adapted to the Brazilian Cerrado, and is also found in Bolivia and Paraguay [1]. Fruits are exploited during harvest in the Brazilian Northeast, without organized cultivation, with the fruits originating from domestic cultivations or even naturally occurring, being sold in street markets and along highways. Its wood is also used in civil construction for internal works such as ceilings, frames, doorframes, floors, and carpentry [2]. Despite being well adapted to the Cerrado, little is known about the water requirements of the species.

Water stress affects plant performance, causing a reduction in the photosystem II quantum efficiency and a reduction in stomatal conductance, Rubisco activity, and photosynthetic rate, which leads to lower plant growth and quality [3,4,5]. The negative effect of these factors can be mitigated by the synthesis of antioxidant and osmoregulatory agents such as proline [6,7].

In this context, several studies have been developed aiming to mitigate the impacts of water stress on plants and understand the patterns and mechanisms of plant responses under this condition. Silicon has been studied as a mitigating agent for the effect of stresses of the most varied kinds. According to [8,9], silicon (Si) plays an important physiological and metabolic role in plants, mitigating the adverse effects of water stress in crops and increasing water use efficiency and antioxidant activity. Salicylic acid (SA) is another compound that has been evaluated for its potential use, as it is a phenolic compound with regulatory action in several physiological processes in plants, and its application is evaluated as a promising strategy to increase the tolerance of crops to biotic, abiotic, and xenobiotic stresses [10,11].

The effects of the application of mitigators on *T. esculenta* growth and physiology are still unknown. Thus, we hypothesized that the application of mitigators reduces the harmful effects of water stress on gas exchange and chlorophyll *a* fluorescence, favors proline synthesis in *T. esculenta* seedlings, and maintains their growth. We also hypothesized that the increase in proline mitigates the effects of water stress, maintaining photosynthetic metabolism and seedling quality. Thus, this study aimed to evaluate the effect of Si and SA application and water stress on the growth and photosynthetic and osmoregulatory metabolism of *T. esculenta* seedlings and their recovery potential after the resumption of irrigation. 

## 2. Results

### 2.1. Gas Exchange

**Figure 1:**
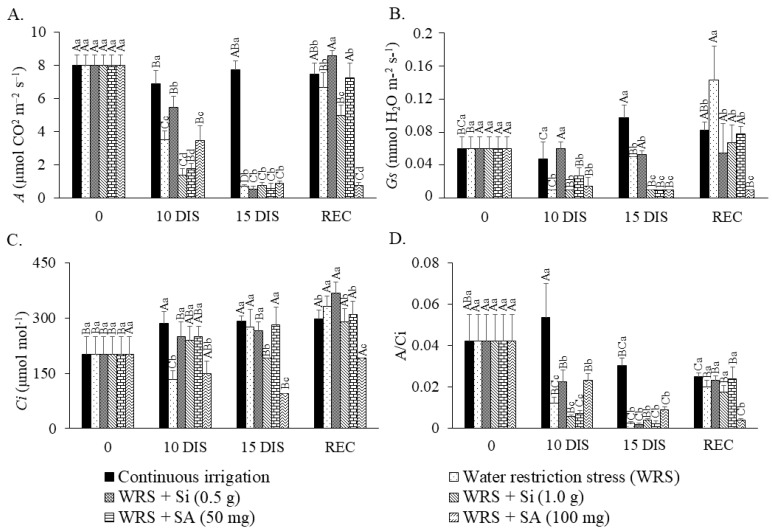
Photosynthetic rate—*A* (**A**), stomatal conductance—*Gs* (**B**), CO_2_ concentration in the intercellular Spaces—*C_i_* (**C**), and carboxylation efficiency—*A*/*C_i_* (**D**) of *Talisia esculenta* Radlk. seedlings during (10 and 15 DIS—days of irrigation suspension) and after (REC) the water restriction period. Equal capital letters (treatment in different evaluation periods) do not statistically differ from each other by the Tukey test (*p* > 0.05), and equal lowercase letters (treatments in the same evaluation period) do not statistically differ from each other by the Scott−Knott test (*p* > 0.05).

The seedlings that received Si application at a dose of 0.5 g showed the highest values of photosynthetic rate (*A*) at 10 days of irrigation suspension, except in comparison to the control. At 15 days of water restriction, all seedlings under stressed treatments showed similar results with *A* values close to one, while the control seedlings maintained high values (Figure 1A).

However, the seedlings that received the lowest doses of Si (0.5 g L^−1^) and SA (50 mg L^−1^) in the recovery period increased *A* values seven days after the resumption of irrigation, which demonstrates the effect of these compounds on the physiological recovery of seedlings. In contrast, the highest doses of Si (1.0 g L^−1^) and SA (100 mg L^−1^) did not have the same effect on the photosynthetic rate at 15 DIS (days of irrigation suspension). The seedlings treated with the highest Si dose recovered only after 28 days, while those treated with the highest dose of SA failed to recover their photosynthetic capacity, even after spending this period under continuous irrigation. Furthermore, we emphasize that the application of 100 g SA L^−1^ on previously stressed seedlings did not contribute to the increase in *A* in the REC. Water deficit caused a reduction in the stomatal conductance (*Gs*) of *T. esculenta* seedlings, especially at 10 DIS. During the period of stress, except for those treated with a silicon dose of 0.5 g L^−1^, which remained at similar values in all periods, the other seedlings had lower values (Figure 1B). In the REC, all the seedlings, except those with 100 mg SA L^−1^, increased *Gs* values, especially those previously stressed without the application of the mitigating products.

Unlike *A* and *Gs*, which showed reduction during the period of water restriction, the internal CO_2_ concentration (*C_i_*) increased during this period, especially in seedlings treated with the two Si doses and the lowest SA dose when compared to the seedlings under deficit without the application of products and those with 100 mg SA at 10 DIS, while at 15 DIS even the seedlings under deficit without Si and SA also increased the values (Figure 1C). In the REC, seedlings of all the treatments, except 100 mg SA L^−1^, increased values.

Thus, we observed that the carboxylation efficiency of Rubisco (*A*/*C_i_*) reduced during the period of water deficit, showing lower values compared to seedlings that remained under irrigation at 10 DIS, highlighting that although the values were reduced compared to the control, the seedlings with 0.5 g Si L^−1^ and 100 mg SA L^−1^ remained higher than the other seedlings subjected to stress in the same period. At 15 DIS, there was a reduction in *A*/*C_i_* values for all seedlings except for the control. However, all treatments showed values similar to those of irrigated seedlings during recovery, except for those treated with 100 mg SA L^−1^ (Figure 1D).

### 2.2. Relative Water Content in the Leaves (RWC)

**Figure 2:**
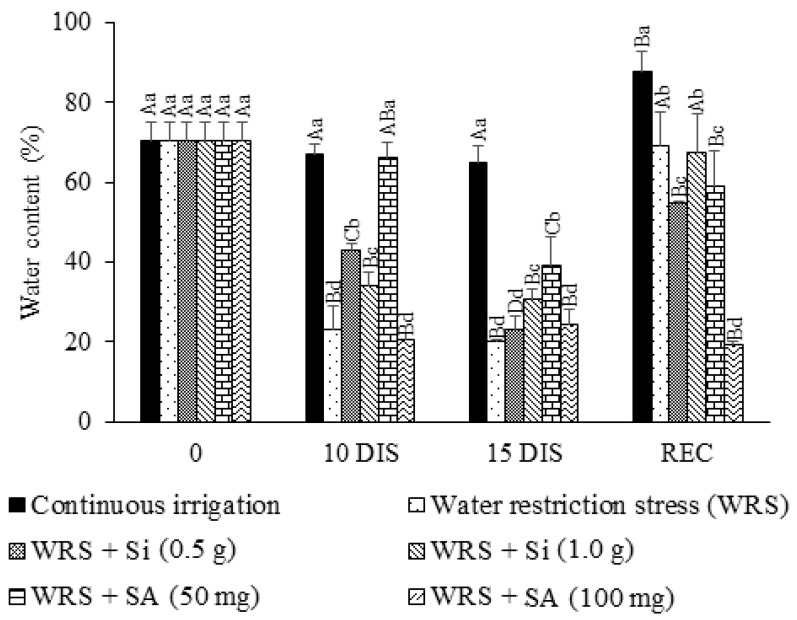
Relative water content of *Talisia esculenta* Radlk. seedlings during (10 and 15 DIS—days of irrigation suspension) and after (REC) the water restriction period. Equal capital letters (treatment in different evaluation periods) do not statistically differ from each other by the Tukey test (*p* > 0.05), and equal lowercase letters (treatments in the same evaluation period) do not statistically differ from each other by the Scott−Knott test (*p* > 0.05).

There was a reduction in the relative water content (RWC) in the leaves of seedlings from all treatments during water restriction, but the seedlings treated with a SA dose of 50 mg L^−1^ only showed this reduction at 15 DIS (Figure 2). SA at 100 mg L^−1^ was the only dose that did not provide an increase during the seedling recovery period. 

### 2.3. Chlorophyll a Fluorescence and Chlorophyll Index

**Figure 3:**
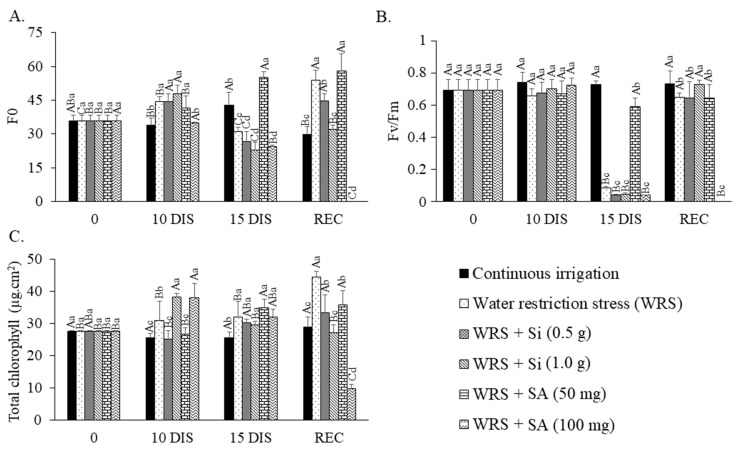
Initial fluorescence—F0 (**A**), photosystem II photochemical potential quantum efficiency—Fv/Fm (**B**), and chlorophyll index (**C**) of *Talisia esculenta* Radlk. seedlings during (10 and 15 DIS—days of irrigation suspension) and after (REC) the water restriction period. Equal capital letters (treatment in different evaluation periods) do not statistically differ from each other by the Tukey test (*p* > 0.05), and equal lowercase letters (treatments in the same evaluation period) do not statistically differ from each other by the Scott−Knott test (*p* > 0.05).

Si and SA applications provided an increase in the initial fluorescence values (F0) up to the 10th day of water restriction, with reduced values on the 15th day. However, seedlings treated with 50 mg L^−1^ of SA showed an increase in F0 throughout the restriction period, whereas the application of 100 mg L^−1^ of SA maintained reduced values throughout this period (Figure 3A). The F0 values increased in seedlings under water stress regardless of Si and SA applications, except in seedlings treated with SA at 100 mg L^−1^. However, F0 decreased at 15 days, except for seedlings that received SA at 50 mg L^−1^. It is important to mention that the F0 values increased again after resuming irrigation, except in seedlings treated with Si at 1.0 g L^−1^.

The photosystem II potential quantum efficiency (Fv/Fm) was reduced only after 15 days of water restriction, and seedlings treated with 50 mg L^−1^ of SA maintained a value significantly higher than all other treatments during this period. However, during recovery, all previously stressed seedlings recovered Fv/Fm values, except those that received SA at 100 mg L^−1^ (Figure 3B).

Water stress caused no permanent damage to Fv/Fm, as the seedlings could recover their values after resuming irrigation regardless of the treatments with the mitigators, except when treated with SA at 100 mg L^−1^.

Si (1.0 g L^−1^) and SA (100 mg L^−1^) doses provided higher total chlorophyll content in the seedlings after 10 days of irrigation suspension (38.1 and 37.9, respectively). However, the lowest Si and SA concentrations during the seedling recovery period were those that provided the highest increase in chlorophyll (Figure 3C).

### 2.4. Leaf and Root Proline Content

**Figure 4:**
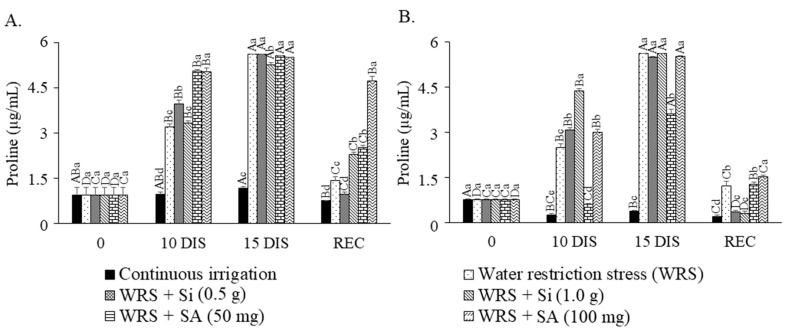
Proline content in leaf (**A**) and root (**B**) of *Talisia esculenta* Radlk. seedlings during (10 and 15 DIS—days of irrigation suspension) and after (REC) the water restriction period. Equal capital letters (treatment in different evaluation periods) do not statistically differ from each other by the Tukey test (*p* > 0.05), and equal lowercase letters (treatments in the same evaluation period) do not statistically differ from each other by the Scott−Knott test (*p* > 0.05).

Proline content increased significantly during both times of water restriction. Leaf proline content increased during the stress period, with silicon treatments maintaining reduced levels for up to 10 days and recovering the seedlings (Figure 4A). The seedlings did not recover the values, remaining high and even reducing the proline content in the leaves after resuming irrigation. Moreover, the lowest SA dose provided the lowest proline content accumulation in the root up to 10 days without irrigation (0.63 µg mL^−1^), while Si favored the highest proline reduction during the recovery period (Figure 4B).

### 2.5. Seedling Quality

**Figure 5:**
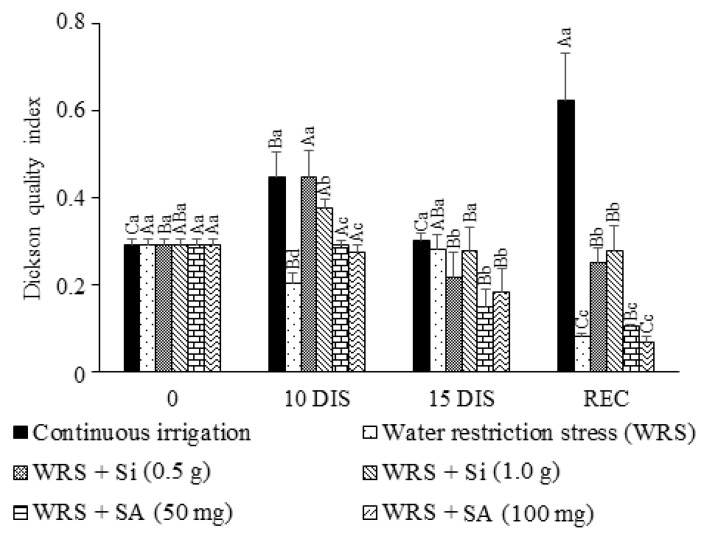
Dickson quality index of *Talisia esculenta* Radlk. seedlings during (10 and 15 DIS—days of irrigation suspension) and after (REC) the water restriction period. Equal capital letters (treatment in different evaluation periods) do not statistically differ from each other by the Tukey test (*p* > 0.05), and equal lowercase letters (treatments in the same evaluation period) do not statistically differ from each other by the Scott−Knott test (*p* > 0.05).

According to the Dickson quality index, Si application provided higher seedling quality up to the 10th day of water restriction. However, Si still provided higher DQI values than treatments with SA, even with reduction at 15 days, with the seedlings treated with 1.0 g L^−1^ showing similar values to those seedlings that remained under irrigation (Figure 5). The seedlings did not recover their quality because they presented significantly lower values than the control.

### 2.6. Ecological Resilience Potential

We observed higher values of the plasticity indices of *A*, *A*/*C_i_*, and F_v_/F_m_ in the seedlings that received the application of 0.5 g of Si compared to the other seedlings (Table 1). In addition, DQI values were lower when compared to biochemical and photochemical metabolisms.

## 3. Discussion

*T. esculenta* seedlings were sensitive to water deficit, as they reduced the photochemical and biochemical characteristics of photosynthesis and greatly increased proline contents. The physiological characteristics of seedlings were better adjusted than the growth characteristics, which demonstrates the faster expression of metabolic responses to water deficit.

Our results confirm our hypothesis that Si and SA at the lowest evaluated doses mitigate the effects of water stress on the photosynthetic rate. However, the hypothesis that an increase in proline is a decisive resource in this mitigation could not be proven by our data.

The positive effect of Si is due to its ability to modulate certain genes related to photosynthesis, also regulating the photochemical process of photosynthesis [12]. This effect of Si on photosynthesis was also pointed out by [4] in seedlings of *Eugenia myrcianthes* Nied. The positive potential of Si in mitigating stress due to water deficit and favoring recovery after resuming irrigation is dose dependent, as the effect is smaller at a higher dose. 

The reduction in stomatal conductance is one of the first effects of water restriction, as the reduction in water availability for plants decreases leaf water potential, which results in loss of turgor in stomatal cells and closure of stomata [13]. The reduction in *Gs* during water deficit has been described by several authors in tree species such as *E. myrcianthes* [3] and *Dipteryx alata* Vogel [5], varying the intensity of the response according to the studied species. A positive effect of leaf application of Si was also observed in *Gs* for lettuce seedlings subjected to stress, with an increase in *Gs* values when increasing silicon doses [14].

Similarly, [5] also observed an increase in *C_i_* values when evaluating Si application in *D. alata* during water stress, which was not followed by photosynthesis, which also showed a reduction, as observed in our study. These results lead us to believe that photosynthesis may also have been limited by enzymatic factors.

Other species showed a similar response to those observed for *T. esculenta*, such as seedlings of *Campomanesia adamantium* (Cambess.) O. Berg [15] and *Campomanesia xanthocarpa* (Mart.) O. Berg. [16] when subjected to water deficit. The lack of water for plants is responsible for damaging the photosynthetic system, reducing the metabolism of the mesophyll, which leads to decrease the activity of Rubisco carboxylase, thus restricting CO_2_ absorption in the chloroplasts and increasing the activity of Rubisco oxygenase, leading to an increase in photorespiration from stomatal closure [15].

We highlight that RWC in the leaves of *T. esculenta* seedlings treated with SA at 100 mg L^−1^ failed to recover turgor even after re-irrigation, showing that these seedlings may have reached the point of permanent wilting, which justifies the pattern response of the evaluated characteristics, with no recovery of photosynthetic metabolism or growth. The reduction in leaf water content during water stress is related to a reduction in soil moisture, which generates a reduction in the water potential of the apoplast, which ends up decreasing the turgor and volume [17].

The beneficial effect of SA suggests that this phytohormone contributed to maintaining leaf turgor even under water deficit conditions, as reported by [3] for *E. myrcianthes* seedlings [3]. The SA plays an important role in the growth of *Olea europaea* L. subjected to drought [18], as its application provides improvements in the adaptive responses of the species in the accumulation of osmolytes, which are crucial to maintaining the leaf turgor, favoring the maintenance of a more favorable hydric state in the dry period and during its recovery.

Evaluating the variables of chlorophyll *a* fluorescence, we observed in the literature that the increase in F0 values is an indication of the stressful cultivation condition [3]. Lower water availability in *C. xanthocarpa* also led to a significant increase in F0 values [19].

The seedlings presented low quantum yields only after 15 days of irrigation suspension, thus showing relevant changes in the potential photochemical efficiency of *T. esculenta* plants subjected to water suspension for a period equal to or higher than this. It indicates that the time of exposure to the stressor, represented here by the irrigation suspension, impaired the photochemical metabolism of the seedlings.

We observed in the literature that plants with Fv/Fm ratio values higher than 0.75 have their photosynthetic apparatus preserved, but values lower than this suggest a reduced photosynthetic potential [9,20,21]. Thus, the evaluation of the variables of chlorophyll *a* fluorescence is important, as seedlings transplanted under field conditions are exposed to stressors such as intense light, drought, and heat [21].

The increase in total chlorophyll in seedlings under water deficit is an atypical result, as the literature records a reduction in photosynthetic pigments in plants subjected to stressful conditions. However, similarly to the results observed for *T. esculenta*, [22] also observed an increase in chlorophyll contents in Barbados nut under water deficit, which suggests resistance of the species to water deficit to enhance the performance of photosynthetic CO_2_ assimilation of plants and maintain their development. However, high SA concentrations can generate a certain impairment of the chloroplast structure. Thus, SA concentrations from 1 mM can cause thylakoid depletion and lumen deformation, which directly affect the total chlorophyll concentration, as suggested by [23].

Proline accumulation and metabolism are associated with mechanisms to prevent abiotic stress in plants and are related to many cellular processes, such as osmotic pressure, energy status, nutrient availability, changes in redox balance, and even defenses against pathogens [24,25].

A review by [26] showed that the increase in the proline biosynthesis rate in chloroplasts during adverse conditions can contribute to the stabilization of the redox balance and the maintenance of homeostasis, dissipating the excess of reducing potential when the electron transport is saturated. Furthermore, the harmful effects of singlet oxygen and hydroxyl radicals on photosystem II in thylakoid membranes can be reduced by proline, as it can protect and stabilize enzymes that scavenge ROS, such as peroxidase, glutathione-S-transferase, superoxide dismutase, and catalase (whose high activity was proven in this experiment for *T. esculenta*, but the results were not presented in this work), and activate alternative detoxification pathways.

However, seedling quality was higher when treated with both Si doses. [26] We observed that proline catabolism in the mitochondria is related to oxidative respiration, which provides energy for resuming growth after stress, but this behavior seems not to have been efficient in *T. esculenta* seedlings. Although the proline content decreased significantly after resuming irrigation, we verified no significant gain in seedling growth. This response can be attributed to the number of days that the seedlings of each treatment took to recover the photosynthetic rate (7 days for Si at 0.5 g L^−1^ and SA at 50 mg L^−1^ and 28 days for Si at 1.0 g L^−1^). Therefore, we believe that if the evaluations were carried out over a longer time, seedling growth could be more expressive.

The Dickson quality index is indicated by [27] as one of the best seedling quality indicators because it provides the pattern and morphological parameters of seedlings, such as shoot height, collar diameter, and dry mass. According to [28], the minimum value of this variable under conditions in which there is higher substrate volume, as is the case in this study, would be around 0.35. However, no treatment reached values equal to or higher than those suggested by the author, except for seedlings that received irrigation throughout the entire experimental period. It shows that this parameter presents variations and, therefore, it is not a sufficient parameter to determine which treatment produces the best seedlings alone, requiring the consideration of other physical and/or biochemistry characteristics of the species.

Although proline content increased under water restriction, this amino acid, considered an important osmoprotective agent and stress mitigator in plants, was not efficient in mitigating the damage caused by water deficit in *T. esculenta* seedlings. In fact, proline could not maintain a low F0 in REC even though it may have contributed to maintaining the Fv/Fm balance at 10 DIS and reduced F0 at 15 DIS. In addition, the increase in proline in both evaluation periods did not act positively to maintain the photosynthetic rate or RWC in the leaves.

Phenotypic plasticity is the ability of genotypes to express different phenotypes in response to environmental conditions. This index varies between 0.00 and 1.00, and values closer to 1.00 represent higher plasticity in the trait [29,30]. In this sense, the application of the products contributes to the induction of stress tolerance, especially at a Si dose of 0.5 g, which favors the increase in antioxidant enzymatic activity and induction of water deficit resistance genes, depending on the dose and target species. On the other hand, although the seedlings treated with 100 mg of SA had high plasticity under this condition, it was not enough to ensure the maintenance of metabolic processes during the period of water deficit and contribute to poststress recovery.

## 4. Materials and Methods

### 4.1. Plant Material, Growth Conditions, and Treatments

Fruits of *T. esculenta* were harvested from parent plants in a remnant area of Cerrado in the Grande Dourados region, MS, Brazil. After manual processing, sowing was conducted in expanded polystyrene trays filled with commercial substrate Carolina Soil^®^ (Pardinho, SP), which were maintained in a nursery under 30% shading and under a 150-micron plastic cover to protect against rainfall. The seedlings were transplanted into 7 L pots filled with clay-textured Oxisol and coarse sand (3:1, *v*/*v*) when they reached an average of 10 cm in height.

The experiment was completely randomized in a 6 × 4 factorial design with three replications consisting of two plants each. The seedlings received the following treatments: (1) irrigation (without stress = 70% of the water retention capacity of the substrate), according to the methodology by [31], (2) irrigation suspension (stress), (3) irrigation suspension + silicon at 0.5 g L^−1^, (4) irrigation suspension + silicon at 1.0 g L^−1^, (5) irrigation suspension + salicylic acid at 50 mg L^−1^, and (6) irrigation suspension + salicylic acid at 100 mg L^−1^. The evaluations were performed at the beginning of the experiment (T0), at 10 (10 DIS) and 15 (15 DIS) days after irrigation suspension, and recovery (REC). The number of days after irrigation suspension (DIS) was determined from pretests to assess when the seedlings would present a photosynthetic rate close to one.

The plants were sprayed to the point of dripping (10 mL of solution per plant). The applications were carried out when the seedlings had an average of 15 cm in height. Potassium silicate (12% Si and density of 1.40 g L^−1^) consisted of the silicon source. After application, the plants were subjected to water restriction, except for the irrigated treatment.

The seedlings were re-irrigated after the evaluation at 15 DIS to assess the recovery, maintaining 70% of the water retention capacity of the substrate. The photosynthetic rate was monitored every two days until the photosynthetic rate of the seedlings that had gone through the water deficit showed a value equal to or higher than 80% of the values found in plants that were not subjected to stress.

### 4.2. Analyses

The following assessments were carried out:

#### 4.2.1. Gas Exchange

Determined always from 8:00 am to 11:00 am using new, completely expanded leaves, using a portable photosynthesis meter and an infrared CO_2_ analyzer (Infra Red Gas Analyzer—IRGA, Model ACD BioScientific Ltd., Hoddesdon, UK) to analyze the following parameters: photosynthetic rate (*A*) (µmol m^2^ s^−1^), stomatal conductance (*Gs*) (mmol H_2_O m^2^ s^−1^), CO_2_ concentration in the intercellular spaces (*C_i_*) (µmol mol^−1^). Using data from *A* and *C_i_*, the carboxylation efficiency (*A*/*C_i_*) was calculated according to that proposed in [9,32]. The saturation point of the photosynthetic system was determined by establishing the flux density value of photosynthetic photons at 700 µmol m^−2^ s^−1^ PPFD.

#### 4.2.2. Relative Water Content in the Leaves (RWC)

Determined in three leaves of each treatment, according to the mathematical expression: RWC = 100 [(fresh mass − dry mass)/(saturated mass − dry mass)]. The leaves were collected in the morning, taken to the laboratory, and cut with cylinders of known area. After weighing the fresh mass, the leaves were placed in a container with distilled water and covered with aluminum foil for 24 h for saturation. After weighing, the saturated disks were dried in a circulation oven to determine the dry mass.

#### 4.2.3. Chlorophyll *a* Fluorescence and Chlorophyll Index

Assessed using an OS-30p portable fluorometer (Opti-Sciences Chlorophyll Fluorometer, Hudson, NY, USA). The leaves were subjected to dark conditions for 30 min. Initial fluorescence (F0) and photosystem II photochemical potential quantum efficiency (Fv/Fm) were considered.

The chlorophyll index was determined using a ClorofiLOG^®^ CFL 1030 portable chlorophyll meter (Falker), with readings on fully expanded leaves.

#### 4.2.4. Leaf and Root Proline Content

Determined by spectrophotometry following the extraction proposed by [33], in which 400 mg of the material was macerated with 10 mL of 3% sulfosalicylic acid and subsequently centrifuged to obtain the supernatant. The determination was performed according to the method by [34], with readings at 520 nm of absorbance.

#### 4.2.5. Seedling Quality

To evaluate the quality of the seedlings, the height, diameter, and dry biomass data of shoots and roots were used, and the Dickson quality index—DQI [35] was calculated.

#### 4.2.6. Ecological Resilience Potential

Estimated through the phenotypic plasticity index (PPI) of *A*, *A*/*C_i_*, Fv/Fm, and DQI, according to the proposal by [30], using the equation: PPI = (M − m)/M, where M is the value of the highest mean and m the value of the lowest mean. We calculated PPI using the highest and lowest values between the seedlings kept under irrigation and those kept under water restriction with and without the application of mitigators. The results were presented only for characterization, not applying statistical analysis.

### 4.3. Statistical Analysis

The data were subjected to analysis of variance and, when significant, the means of evaluation periods were compared by the Tukey test (*p* ≤ 0.05) and the treatments by the Scott-Knott test (*p* ≤ 0.05) using the software SISVAR [36].

## 5. Conclusions

Our results show that although *T. esculenta* is found in places with an arid climate, its seedlings were very sensitive to water deficit, with a significant reduction in the photosynthetic rate, stomatal conductance, Rubisco carboxylation efficiency, relative water content in the leaves, and photosystem II potential quantum efficiency, and increased proline content. The seedlings recovered the photosynthetic rate in just seven days after resuming irrigation when treated with Si at 0.5 g L^−1^ and SA at 50 mg L^−1^. 

The seedlings showed a significant increase in the other evaluated characteristics regardless of the application of these mitigators, although they took time to recover the photosynthetic rate or did not recover the values of the other evaluated characteristics in most treatments.

The salicylic acid dose of 100 mg L^−1^ is not recommended because it did not allow the recovery of seedling characteristics.

## Figures and Tables

**Table 1:** Phenotypic plasticity index (PPI) for photosynthetic rate (*A*), carboxylation efficiency (*A/C_i_*), photosystem II photochemical potential quantum efficiency (F_v_/F_m_), and Dickson quality index (DQI) in plants grown under water restriction stress without or with doses of silicon and salicylic acid.

Treatments	PPI (0.00 a 1.00)			
	*A*	*A*/*C_i_*	F_v_/F_m_	DQI
Water restriction stress (WRS)	0.909061	0.900543	0.883402	0.069024
WRS + Si (0.5 g)	0.931068	0.923442	0.948788	0.278752
WRS + Si (1.0 g)	0.902589	0.851954	0.938272	0.278752
WRS + SA (50 mg)	0.925566	0.917749	0.190672	0.499342
WRS + SA (100 mg)	0.888673	0.663373	0.947417	0.388007

## Data Availability

The data presented in this study are available in the graphs and tables provided in the manuscript.
